# City Drug Stores

**Published:** 1853-09

**Authors:** 


OUR CITY DRUG STORES.

We would call the attention of the medical profession to the advertisements, upon the cover of this journal, of our excellent Drug Establishments. No city in this section of the country, can boast of more elegant stores, of better assortments, or of purer medicines. There is a laudable emulation to procure every article required by the practitioner, or demanded by the wants of the afflicted; to furnish select and pure remedies, and in every respect to keep pace with new improvements.

Mr. Davis, on Water street, is an old and experienced druggist, and brings to his business the reputation of years.

Mr. Ayers, on the corner of Third and Main streets, has an establishment which, for taste and elegance, is rarely excelled in any city; the character of his selections and the extensiveness of his assortment, are unsurpassed. He has almost grown up in a drug store and is familiar with all its details. He occupies the whole building, and one who wanders through his ample premises, can hardly refrain from verging upon the conclusion, that such an employment should rank among the learned professions!

Messrs. Gilmore & McKenny, nearly opposite, have fitted up anew, in a style exceedingly neat and in elegant taste. They also are supplied with every variety of drugs and medicines, and pay special attention to the purity and elegance of their articles. Long and careful devotion to their business enables them to excel.

W. H. Farner & Co. are also furnished with the usual supply of the trade, and both Mr. F. and his partner, Mr. Taunton, are skillful druggists, and sustain a most favorable reputation.

The excellent and well sustained reputation of all these Houses, serves to render Keokuk the great center of this department of trade, for the State, and brings to this city an extensive patronage which deserves only to be enlarged.

